# Protective effects of a compound herbal extract (Tong Xin Luo) on free fatty acid induced endothelial injury: Implications of antioxidant system

**DOI:** 10.1186/1472-6882-8-39

**Published:** 2008-07-14

**Authors:** Lin Zhang, Yiling Wu, Zhenhua Jia, Yun Zhang, Hu Ying Shen, Xing Li Wang

**Affiliations:** 1 Michael E. DeBakey Department of Surgery, Baylor College of Medicine, Texas Heart Institute, Houston, Texas, USA; 2 Research Institute of Integrated Traditional Chinese Medicine and Western Medicine of Hebei, Hebei, PR China; 3 Key Laboratory of Cardiovascular Remodeling and Function Research, Chinese Ministry of Education and Chinese Ministry of Health, Shandong University Qilu Hospital; Jinan, PR China

## Abstract

**Background:**

Tong-Xin-Luo (TXL) – a mixture of herbal extracts, has been used in Chinese medicine with established therapeutic efficacy in patients with coronary artery disease.

**Methods:**

We investigated the protective role of TXL extracts on endothelial cells injured by a known risk factor – palmitic acid (PA), which is elevated in metabolic syndrome and associated with cardiovascular complications. Human aortic endothelial cells (HAECs) were preconditioned with TXL extracts before exposed to PA for 24 hours.

**Results:**

We found that PA (0.5 mM) exposure induced 73% apoptosis in endothelial cells. However, when HAECs were preconditioned with ethanol extracted TXL (100 μg/ml), PA induced only 7% of the endothelial cells into apoptosis. Using antibody-based protein microarray, we found that TXL attenuated PA-induced activation of p38-MAPK stress pathway. To investigate the mechanisms involved in TXL's protective effects, we found that TXL reduced PA-induced intracellular oxidative stress. Through AMPK pathway, TXL restored the intracellular antioxidant system, which was depressed by the PA treatment, with an increased expression of thioredoxin and a decreased expression of the thioredoxin interacting protein.

**Conclusion:**

In summary, our study demonstrates that TXL protects endothelial cells from PA-induced injury. This protection is likely mediated by boosting intracellular antioxidant capacity through AMPK pathway, which may account for the therapeutic efficacy in TXL-mediated cardiovascular protection.

## Background

Coronary artery disease (CAD), as a multifactorial disease, is the consequence of interactions between modern life style and susceptible genes. Although significant progress has been made in the development of preventive and therapeutic strategies in managing CAD, the CAD prevalence appears to have reached the plateau and remains the major cause of mortality and morbidity in most developed and developing nations. Advent of statin class drugs – HMG-CoA reductase inhibitor, has made cholesterol reduction readily achievable. However, hypercholesterolemia explains less than 50% of CAD risk. Other risk factors including cigarette smoking, metabolic syndrome and arterial wall specific risks explain a large proportion of the unexplained pathologies.

Among established risk factors for CAD, metabolic syndrome is one of the modern day epidemics and is characterized by increased levels of circulating nonesterified free fatty acids (FFAs). FFAs provide an important energy source as well as acting as signaling molecules in various cellular processes. However, a chronic elevation of FFAs as seen in metabolic syndrome is strongly associated with cardiovascular complications [1,2]. Although FFAs-induced metabolic insulin resistance and sustain hyperglycemia may be a mechanism, excessive FFAs may also have direct effects on vascular functions [3]. A significant relationship between FFA levels and baseline systolic and diastolic blood pressure has been reported [4]; and inappropriate elevation of plasma FFAs is associated with impaired endothelium-dependent vasodilation in both healthy and insulin resistant human subjects and animals [5,6]. Elevation of FFAs also induces inflammation in healthy subjects [7-9] and in endothelial cells [10,11]. Additionally, high FFA levels are significantly associated with stroke[12], myocardial infarction [12] and sudden death [13]. Thus, FFAs may play a proximal pathophysiological role and serves as a potentially causative link between obesity, type 2 diabetes and cardiovascular diseases [14-18]. Among FFAs, palmitic acid (PA) is a saturated fatty acid and appears to promote endothelial apoptosis, thereby increases the risk of vascular diseases [10,19,20]. Apoptosis is a universal biological phenomenon regulating cell proliferation, differentiation and specialization [21,22]. Dysregulated apoptotic processes, either genetically programmed or environmentally triggered, can result in a range of abnormalities in every body system. Excessive endothelial apoptosis is generally regarded as atherogenic and thrombogenic [23-26].

Despite the significant progress in the understanding of endothelial dysfunction and vascular disease, no pharmacologically active agent has been developed to therapeutically modulate this connection. Currently employed pharmaceutical development strategies appear to be stagnant in discovering new drug with efficacy as powerful as the statins. On the other hand, traditional medicine has been practiced for hundreds and thousands of years in some communities, such as American Indians or Chinese. One of the major therapeutic modalities is herbal medicine with different mixing formulas in treating various clinical conditions. With availability of modern technologies, preparation of the herbal medicine has also evolved and some herbal compound extracts being developed and used clinically with success. Among many of the compound herbal extracts, Tong-Xin-Luo (TXL) was developed 2 decades ago for the treatment of CAD (registered in State Food and Drug Administration of China). TXL is a mixture of herbal extracts and has been successfully used in thousands of patients with chronic CAD in reducing the occurrence of acute coronary events or sudden death. TXL was extracted, concentrated and freeze-dried from a mixture of ginseng, red peony root, borneol and spine date seed. One therapeutic course is normally prescribed as 2–4 capsules 3 times daily for 4 weeks. Clinical trials have shown that standard medical treatment complemented with TXL is more effective than standard therapy alone in reducing infarct size, recovery time and improvement in ventricular function in patients with acute coronary syndrome [27,28]. The beneficial effects are further demonstrated in animal models [28].

In this study, we investigated molecular targets that may be responsible for TXL mediated endothelial protection. It is of note that TXL as compound extracts contain multiple active components that may be responsible for the observed therapeutic effects. Our strategy is to use the extracts that have proven clinical benefits to identify molecular targets that are influenced by the TXL. We challenged the TXL-preconditioned endothelial cells with PA and explored the molecular changes in these endothelial cells. We found that TXL protected PA-induced endothelial damage by initiating AMPK-mediated activation of thioredoxin (Trx) antioxidant system.

## Methods

### Preparation of Fatty Acid-Albumin Complexes, TXL and Endothelial Treatment

Saturated PA was used in this study. Lipid-containing media were prepared by conjugation of PA with bovine serum albumin (BSA) using a modification of the method described previously [29]. Briefly, PA was first dissolved in ethanol at 200 mM, and then combined with 10% FFA-free low endotoxin BSA to final concentrations of 1–5 mM. The pH of the solution was adjusted to approximately 7.5, and the stock solution was filter-sterilized and stored at -20°C until use. Control solution containing ethanol and BSA was prepared similarly. Working solutions were prepared fresh by diluting stock solution (1:10) in 2% FCS-EBM (fetal calf serum-endothelial cell basic medium).

In order to investigate the protective effects by the TXL, we dissolved the TXL in three different types of solvents including phosphate buffered saline (PBS), dimethyl sulfoxide (DMSO) and ethanol. We prepared TXL solution by mixing 100 mg TXL in 10 ml PBS or DMSO or ethanol as a stock solution (10 mg/ml). After the vortex mix, the solution was then filtered through a 0.2 micron filter, which was then aliquoted and stored at -20°C before use.

Primary human aortic endothelial cells (HAECs) (Cell Applications, San Diego, CA) were cultured in endothelial cell growth medium-2 (EGM-2) medium (Cambrex, East Rutherford, NJ) containing EBM, hydrocortisone, FGF-B, VEGF, IGF-1, EGF, ascorbic acid, GA-1000, heparin, and 2% FBS in 5% CO_2 _at 37°C. Cells cultured up to five passages were first grown to 90% confluence before exposed to PA (0 – 0.5 mM) or TXL (10 – 100 μg/ml) for 24 hours. In order to test the TXL-mediated endothelial protection, we first preconditioned the HAECs with TXL for 30 min before they were exposed to PA for additional 24 hours. These cells were then tested for apoptosis or subjected to protein extraction for Western blot.

### siRNA-induced Gene Silencing

Silencing gene expression was achieved using the siRNA technique. AMPK siRNA was purchased from Ambion (Austin, Texas). Transfection of HAECs with siRNAs was carried out using LipofectAMINE™ 2000 (Invitrogen, Carlsbad, California), according to the manufacturer's instruction. Transfected cells were then treated with PA or TXL at the designated concentrations for the time periods indicated in the text.

### Detection of Apoptosis

We used terminal deoxynucleotidyltrasnferase-mediated dUTP nick-end labeling (TUNEL) assay to measure the endothelial apoptosis. The TUNEL was performed using the in situ cell detection kit following the manufacturer's instructions (BD Biosciences). In brief, after designated treatments, HAECs grown on gelatin-coated coverslips were washed twice by PBS, and fixed by 4% paraformaldehyde solution in PBS for 15 min at room temperature. Coverslips were then washed with PBS and permeabilized in 0.2% Triton X-100/PBS for 10 min. Each coverslip was added 50 μl of the TUNEL reaction mixture and incubated in a dark humidified chamber for 1 h at 37°C. The reaction was terminated by adding 2 #215; SSC and incubated at room temperature for 15 min. The DNA dye DAPI (4'6' Diamidino-2-phenylindole dihydrochloride) was used to label the nuclei at the concentration 0.1 μg/ml for 30 min. The slides were examined with a Leica DMLS Epifluoresence microscope (200× magnification). The data were analyzed with the Image-Pro Plus V4.5 software (Media Cybernetics, Inc).

### Detection of Intracellular ROS Levels

Intracellular ROS level was determined using the oxidant-sensitive fluorogenic probe CM-H_2_DCFDA (5-(and-6)-chloromethyl-2',7'-dichlorodihydrofluorescein diacetate, acetyl ester) from Invitrogen, Carlsbad, California. HAECs were treated with FFAs for 24 hours with or without TXL preconditioning and washed with PBS. Treated cells were incubated with 5 μM DCFH-DA in serum free medium for 30 minutes at 37°C. Fluorescence was detected by a fluorescent microscope, and its intensity in individual cells was analyzed.

### Western Blot

Treated cells were collected and lysed as described previously [30]. Protein samples (15 μg per lane) were subjected to SDS-polyacrylamide gel electrophoresis and transferred to PVDF membranes. The membranes were blocked, treated with primary antibody, washed, and then incubated with the secondary horseradish peroxidase-labeled antibody. Bands were visualized with Enhanced Chemiluminescence (Amersham Biosciences, Piscataway, NJ). The data shown were representative of three experiments. Trx, thioredoxin interacting protein (Txnip) and AMPK antibodies were purchased from Cell Signaling (Beverly, MA).

### Antibody-based Protein Microarray

In the analysis of protein microarray, we compared protein profiles in HAECs treated with TXL/PA to PA alone; HAECs treated with PA alone to culture medium control; HAECs treated with TXL/PA to 1% ethanol (as the vehicle amount used in the TXL treatment); HAECs treated with 1% ethanol to culture medium blank control. Crude cell/tissue lysates were prepared in the lysis buffer (20 mM MOPS, 60 mM β-glycerophosphate, 5 mM EDTA, 2 mM EGTA, 1 mM Na_3_VO_4_, 30 mM NaF, 0.5% Nonidet P-40, and 1 mM DTT, supplemented with 1 mM PMSF, 10 μM leupeptin, 4 μg/ml aprotinin and 5 μM pepstatin A) as stipulated by Kinexus (Kinexus Bioinformatics Corporation, Vancouver, British Columbia, Canada). Fifty micrograms of protein lysate were labeled with a fluorescent dye at a concentration of 2 mg/ml, and unincorporated dye molecules were removed by ultrafiltration. Purified labeled proteins from the control and its correspondingly treated sample were incubated simultaneously on a Kinex™ antibody microarray side by side (Kinexus). Each Kinex antibody microarray has 2 identical fields of antibody grids containing 608 antibodies each that target various cell signaling proteins (Additional file 1). After probing, arrays were scanned using a ScanArray scanner (Perkin Elmer, Wellesley, USA) with a resolution of 10 μm, and the resulting images were quantified using ImaGene (BioDiscovery, El Segundo, CA). We regarded proteins with differences of 2.0-fold or more as significant.

## Results

### TXL-Mediated Protection against PA-Induced Endothelial Apoptosis

**Figure 1:**
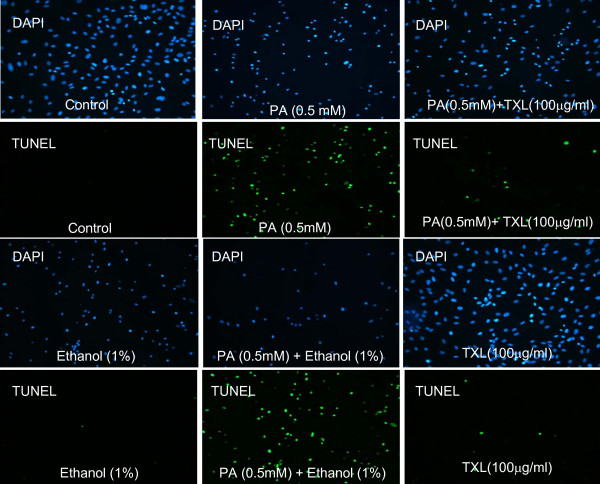
**TXL-mediated protection against PA-induced endothelial apoptosis**. PA at the concentration of 0.5 mM was used to treat endothelial cells with or without TXL preconditioning. TXL preconditioning was carried out by adding ethanol extracted TXL (100 μg/ml) to cultured endothelial cells 30 min prior to the exposure of PA. Apoptosis was detected using TUNEL assay in which DAPI stained nuclei blue. Percentages of apoptosis were calculated as the number of nuclei with positive TUNEL stain to the total number of nuclei (DAPI stain). Cells were visualized under fluorescence microscope (magnification × 200).

Dysregulated endothelial apoptosis plays an important role in endothelial dysfunction, vascular inflammation, pathological thrombus formation and atherosclerosis. We therefore examined whether TXL had any protective effect on endothelial apoptosis which was detected by the TUNEL assay. As shown in Figure 1, PA induced apoptosis in a dose dependent manner; PA treatment (0.5 mM) resulted in nearly 70% cell death. However, in HAECs preconditioned with 100 μg/ml of TXL, PA exposure (0.5 mM) resulted in only 7% apoptosis. These findings indicate that the ethanol extracted TXL had a clear anti-apoptotic effect on PA-induced endothelial apoptosis. Ethanol alone had minimal protection (Fig. 1A). The PBS extracted TXL had no protection on PA-induced endothelial apoptosis (data not shown). While DMSO-extracted TXL also showed the protective effects, the protection demonstrated in cells treated with DMSO alone accounted for most of the protection (data not shown). Therefore, we carried out rest of the studies using ethanol extracted TXL.

**Figure 2:**
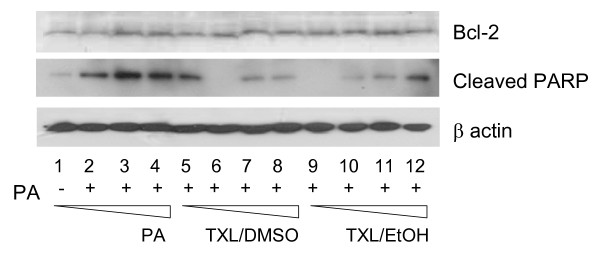
**Changes in apoptotic pathways in TXL and/or PA treated endothelial cells**. Detection of cleaved PARP in cells treated with increasing doses of PA (0, 0.3, 0.4 and 0.5 mM), TXL dissolved in DMSO (0, 10, 50 and 100 (g/ml) or TXL dissolved in ethanol (0, 10, 50 and 100 (g/ml). Bcl-2 was detected with the specific antibody and beta-actin was used as the loading control.

In the process of investigating the mechanisms of TXL-mediated protection on PA-induced endothelial apoptosis, we found that TXL had no effects on PA-induced caspase-3 activation (data not shown) nor the expression of Bcl-2 (Fig. 2). However, TXL significantly attenuated the PA-induced PARP elevation (Fig. 2), which is one of the last steps in apoptotic nuclear DNA cleavage.

### TXL Mediated Protein Changes in PA Treated Endothelial Cells

In order to explore protein changes that may be responsible for TXL-mediated protection, we used antibody microarray to interrogate 608 proteins (Additional file 1). We compared the protein profiles in cells treated with TXL/PA, PA, ethanol and control medium for the fluorescence intensity as illustrated in the Additional file 2 (Figure A and B). More than 90 proteins were decreased by the TXL preconditioning in PA-exposed endothelial cells (Additional file 3). The reduction in these proteins was clearly consistent with the protective effects on endothelial cells. For example, PA treatment activated p38 MAPK (as marked by both pan-specific increase in phosphorylated p38 and phosphorylated p38 at T180 and Y182). The TXL preconditioning, however, induced 2.7 to 8.1-fold reduction in p38 (Additional file 3). This pattern of action was further demonstrated by the reduction in MEKK2, MEKK4, MEK3 and MEK6. As a pro-apoptotic factor, PA also increased phospho-Ser392-p53 – a tumor suppressor protein inducing cell death. Endothelial cells preconditioned with TXL had reduced p53 by nearly 6.0-fold. The anti-apoptotic effect was further illustrated by the reduction in PARP1 that was activated by the PA treatment. Furthermore, PA as an inflammatory trigger also activated PKC and PKA kinase systems, both of which were suppressed by the TXL preconditioning to as much as 15-fold. A similar effect was also observed for RIPK1 and RSK kinases, which are all part of proinflammatory responses.

More than 100 proteins were increased more than 2-fold by the TXL treatment (Additional file 3). Among the proteins increased by the TXL treatment, JAK1-Stat proteins appeared to be prominent with more than 2.0-fold increases comparing to the HAECs treated with PA only. The JAK1-STAT is a well-recognized cell survival signaling pathway. Activation of the pathway by TXL could be responsible for the TXL-mediated anti-apoptotic effects. In corresponding with the activation of this pathway, the Smad associated TGFβ pathway was also activated by the TXL with more than 2-fold increase in phospho-Smad 2/3. Furthermore, the AMPK regulatory subunit was elevated by the TXL pretreatment (>2.0-fold). The cell growth signaling pathways including Rb gene and arrestin beta 1 were also elevated. All these changes are consistent with the anti-apoptotic effect. In line with the pro-growth capacity, transcription factors including eIF2a, eIF4E, JNK, Jun and EGFR were upregulated by the TXL treatment as well.

### Effects of TXL on PA-induced Oxidative Stress

**Figure 3:**
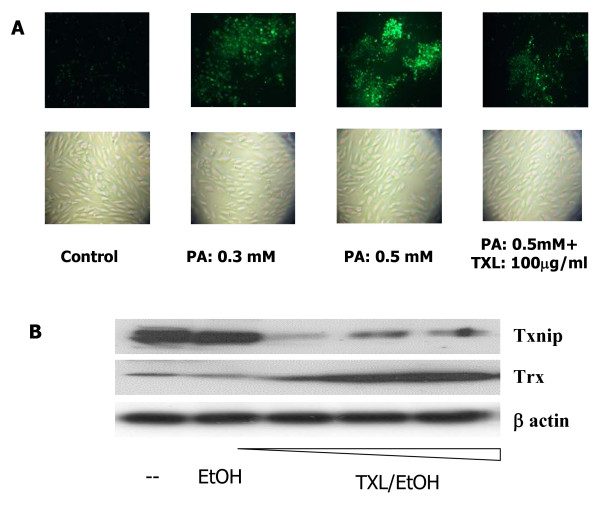
**Effects of TXL on PA induced ROS production in endothelial cells**. **A**: Endothelial cells were treated with different doses of PA (0, 0.3 and 0.5 mM) with or without TXL preconditioning. Treated cells were incubated with oxidant-sensitive fluorogenic probe CM-H_2_DCFDA for the detection of ROS. The stained cells were visualized under fluorescence microscope (magnification × 200). Brightfield images were also taken for comparison. **B**: Effect of TXL (0, 10, 50 and 100 (g/ml) on protein expression of Txnip and Trx by Western blot. Beta-actin was used as a loading control.

Previous studies suggest that excess PA induces oxidative stress. We therefore investigated whether TXL had any effect on PA-induced oxidative stress. As expected, the superoxide production was increased in endothelial cells exposed to PA (Fig. 3A). However, in endothelial cells that were first preconditioned with TXL before the PA treatment, the superoxide production was significantly reduced (Fig. 3A).

In order to investigate how TXL hindered the ROS production induced by the PA, we examined one of the major intracellular antioxidant pathways. Trx is the key component of the intracellular antioxidant system, which protects proteins from oxidative damage by donating -SH group. Our experiment showed that TXL significantly increased the expression of Trx (Fig. 3B). At the same time, TXL treatment also reduced the expression of Txnip (Fig. 2B), which is the antagonist of the Trx. These findings suggest that one of the pathways mediating the protective effects of the TXL on endothelial cells is by upregulating intracellular antioxidant system.

### Involvement of AMPK Pathway in TXL-Mediated Protection

**Figure 4:**
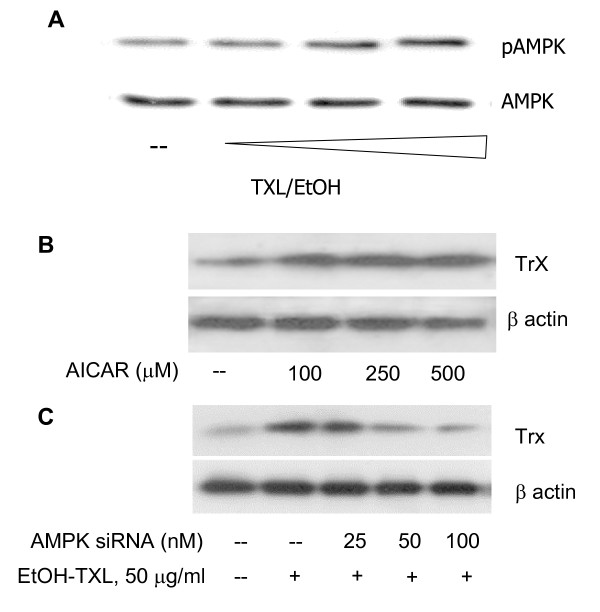
**Western blot detection of TXL induced AMPK activation and Trx expression in endothelial cells**. **A**: While TXL (0, 10, 50 and 100 (g/ml) had on effect on the protein levels of total AMPK, it significantly increased the levels of phospho-ser172-AMPK. **B**: Changes of Trx in endothelial cells treated with AMPK activator AICAR at increased doses. **C**: Effect of AMPK knockdown on Trx expression in endothelial cells treated with TXL. AMPK knockdown abolished the upregulatory effect of TXL on Trx.

**Figure 5:**
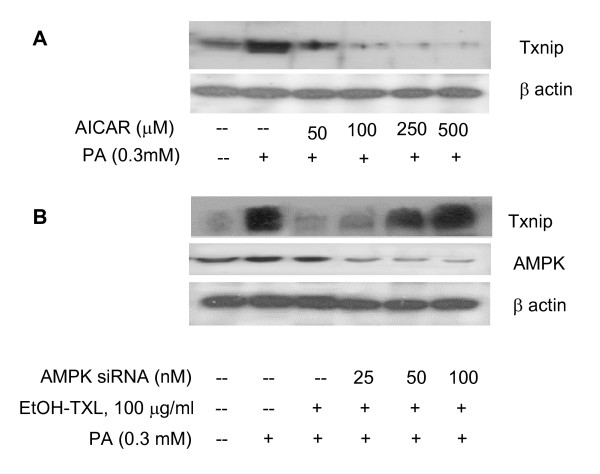
**Protein levels of Trx antagonist Txnip in endothelial cells with different treatments**. **A**: Txnip protein changes in endothelial cells treated with AMPK activator AICAR with or without co-treatment of PA. While PA upregulated the Txnip level, the activation of AMPK pathway using AICAR reduced Txnip levels. **B**: Role of AMPK in the down-regulatory effect of TXL on Txnip expression in cells treated with or without PA. While PA (0.3 mM) clearly increased the Txnip expression, TXL (100 (g/ml) preconditioning abolished the PA effect. However, AMPK silence induced by the gene specific siRNA (middle panel) appeared to diminish the effect of TXL.

Since AMPK pathway is involved in PA-related oxidative stress [2,31,32], we then examined whether AMPK was involved in TXL-induced Trx upregulation. As shown in Figure 4A, TXL directly increased the amount of phospho- Ser172-AMPK while the total AMPK remained unchanged. The activation of AMPK, as demonstrated using the specific AMPK activator AICAR resulted in an increased expression of Trx (Fig. 4B). Suppression of AMPK with gene specific siRNA resulted in a significant reduction in Trx expression and indeed blocked the TXL-mediated Trx elevation (Fig. 4C). In contrast, while PA upregulated the Txnip expression (Fig. 5A), TXL blocked this effect and resulted in suppression of Txnip. The TXL-mediated Txnip downregulation appeared to be mediated through the AMPK pathway as well since AMPK silence with gene specific siRNA attenuated the effects of TXL on Txnip (Fig. 5B). We therefore suggest that TXL activates Trx antioxidant system through AMPK pathway.

## Discussion

Over more than 2 decade clinical administration, TXL has proven clinical beneficial effects in CAD patients. Current study suggests that the protective effect by TXL is likely mediated through the activation of intracellular thioredoxin antioxidant system. AMPK pathway regulates oxidative metabolism of fatty acids and glucose [32,33]. Our study demonstrates that TXL attenuates the damaging effects of PA via activating the AMPK pathway. If AMPK activation triggered by the exposure to TXL is not confined to endothelial cells, it would suggest a potentially new application of TXL in regulating glucose metabolism and insulin sensitivity.

Human subjects, like all other aerobic organisms, are constantly challenged by free radicals or reactive oxygen species (ROS) that are produced during normal metabolism [34-36]. On the other hand, cells also possess a battery of antioxidant systems to specifically counterbalance individual species of ROS. Among available intracellular antioxidants, thioredoxin system is one of the most important intracellular antioxidant systems [37-41] that maintains the reduced status of peroxiredoxin for the reduction of H_2_O_2_; suppresses signaling protein ASK1 from activating p38 MAPK-mediated apoptosis pathway; regulates expression of stress proteins including transcription factors.

In the conditions of metabolic syndrome, FFAs are elevated; excessive ROS is produced during the oxidation metabolism of FFA. In excess, ROS and their byproducts are capable of causing oxidative damage and cytotoxic to endothelial cells. ROS can promote endothelial apoptosis; [42]; increase the endothelial permeability, which allows atherogenic LDL accumulating in the sub-endothelial space; stimulate endothelial cell production of adhesion molecules rendering vascular wall pro-thrombotic and pro-atherogenic [43]. Some antioxidants were shown to successfully attenuate the oxidative stress induced endothelial dysfunction [44-48]. However, no antioxidants are shown to be therapeutically effective in protecting endothelial cells from injury. Findings of our study in illustrating the effects of TXL on the protection of Trx system offer a possibility to discover compounds that could be potentially used pharmacologically to energize intracellular antioxidant system.

In this study, we used high-throughput antibody microarray approach to systemically discover the intracellular systems that are activated by the TXL. The results consistently indicated the attenuation of the stress signaling pathways by TXL; some of these changes are confirmed by the Western blot and functional studies. Our study suggests that the high throughput approach is an effective tool when no prior knowledge is established for the biological effects of a compound.

One of the major limitations of the current study is the compound mixture of the TXL extracts, in which it is not known which component(s) is responsible for the observed protective effects on PA-induced endothelial damage. Like all other herbal medicines, there are also no biomarkers to test the effective plasma levels in relation to the drug doses used in the patients. The doses used in the current study were based on previous experiments and dose-dependent effects observed in our laboratory. It should be acknowledged that as a part of continuing efforts in discovering active components with specific molecular targets, our next project is to fractionate the individual components of the compound and to evaluate the protective effects of each individual component. This strategy has a higher chance of success in discovering functional molecules since it is based on an extract mixture with established clinical benefits demonstrated over decades. It has the advantage over the approaches that are developed on the basis of *in vitro *effects. These active molecules discovered through *in vitro *experiments or *in vivo *animal models are frequently found either without effects when applied to humans or with unacceptable side effects. To discover effective molecules from the compounds that have proven clinical effect may be a cost-effective alternative drug discovery strategy.

## Conclusion

In summary, we have found that clinical efficacy in TXL mediated cardiovascular protection is at least partly mediated through the activation of intracellular thioredoxin antioxidant system, which is the consequence of AMPK pathway activation. While further studies are needed to discover the active components of the TXL compound mixture that are responsible for the protection, current study identify the intracellular antioxidant system that can be targeted when searching for the active components.

## Abbreviations

4'6' Diamidino-2-phenylindole dihydrochloride: DAPI; 5'AMP-activated protein kinase: AMPK; Bovine serum albumin: BSA; Coronary artery disease: CAD; Dimethyl sulfoxide: DMSO; Dithiothreitol: DTT; Endothelial cell growth medium-2: EGM-2; Epidermal growth factor receptor: EGFR; Epidermal growth factor: EGF; Ethylene glycol tetraacetic acid: EGTA; Ethylenediaminetetraacetic acid: EDTA; Fetal calf serum-endothelial cell basic medium: FCS-EBM; Fibroblast growth factor B: FGF-B; Free fatty acids: FFAs; Human aortic endothelial cells: HAECs; Human aortic endothelial cells: HAECs; Insulin-like Growth Factor-1: IGF-1; Janus kinases: JAKs; Jun N-terminal kinase: JNK; MAP kinase kinase kinase: MEKK; Mitogen-Activated Protein Kinase: MAPK; Palmitic acid: PA; Poly (ADP-ribose) polymerase: PARP; Polyvinylidene Fluoride: PVDF; Pphosphate buffered saline: PBS; Protein kinase C: PKC; Reactive oxygen species: ROS; Receptor-interacting serine/threonine kinase: RIPK; Ribosomal s6 kinase: RSK; Signal Transducers and Activators of Transcription: STAT; Terminal deoxynucleotidyltrasnferase-mediated dUTP nick-end labeling: TUNEL; Thioredoxin interacting protein: Txnip; Thioredoxin: Trx; Tong-Xin-Luo: TXL; Transforming growth factor beta: TGF-β; Vascular endothelial growth factor: VEGF.

## Competing interests

The authors declare that they have no competing interests.

## Authors' contributions

LZ performed the experiments and data analyses; YW conceived the project design, data interpretation and analyses; ZJ participated in the project design and coordination, data analyses and manuscript revision; YZ conceived the project, experimental design, data analyses and result presentation; YHS responsible for experimental design, data interpretation, results analyses and manuscript writing; XLW responsible project design, results analyses and manuscript preparation and revision. All authors read approved the final manuscript.

## Pre-publication history

The pre-publication history for this paper can be accessed here:



## Supplementary Material

Additional file 1 Protein identification in the Kinex antibody microarray that contains 608 antibodies each that target various cell signaling proteins. Click here for file

Additional file 2 Fluorescence images of the Kinex antibody microarray in detecting changes of protein expression in cultured endothelial cells treated with ethanol or control medium (Figure A), PA and/or TXL (Figure B). Click here for file

Additional file 3 Protein expressions with significant changes in cultured human endothelial cells exposed to PA with or without TXL and vehicle controls detected by Kinex antibody microarray. Click here for file
