# Supplementary material for: Protective effects of a compound herbal extract (Tong Xin Luo) on free fatty acid induced endothelial injury: Implications of antioxidant system
Source: BMC Complement Altern Med. 2008 Jul 14;8:39. doi: 10.1186/1472-6882-8-39 (PMC2478673; doi:10.1186/1472-6882-8-39)
Supplement: Additional file 2 — Fluorescence images of the Kinex antibody microarray in detecting changes of protein expression in cultured endothelial cells treated with ethanol or control medium (Figure A), PA and/or TXL (Figure B). [file 1472-6882-8-39-S2.ppt]

## Slide 1
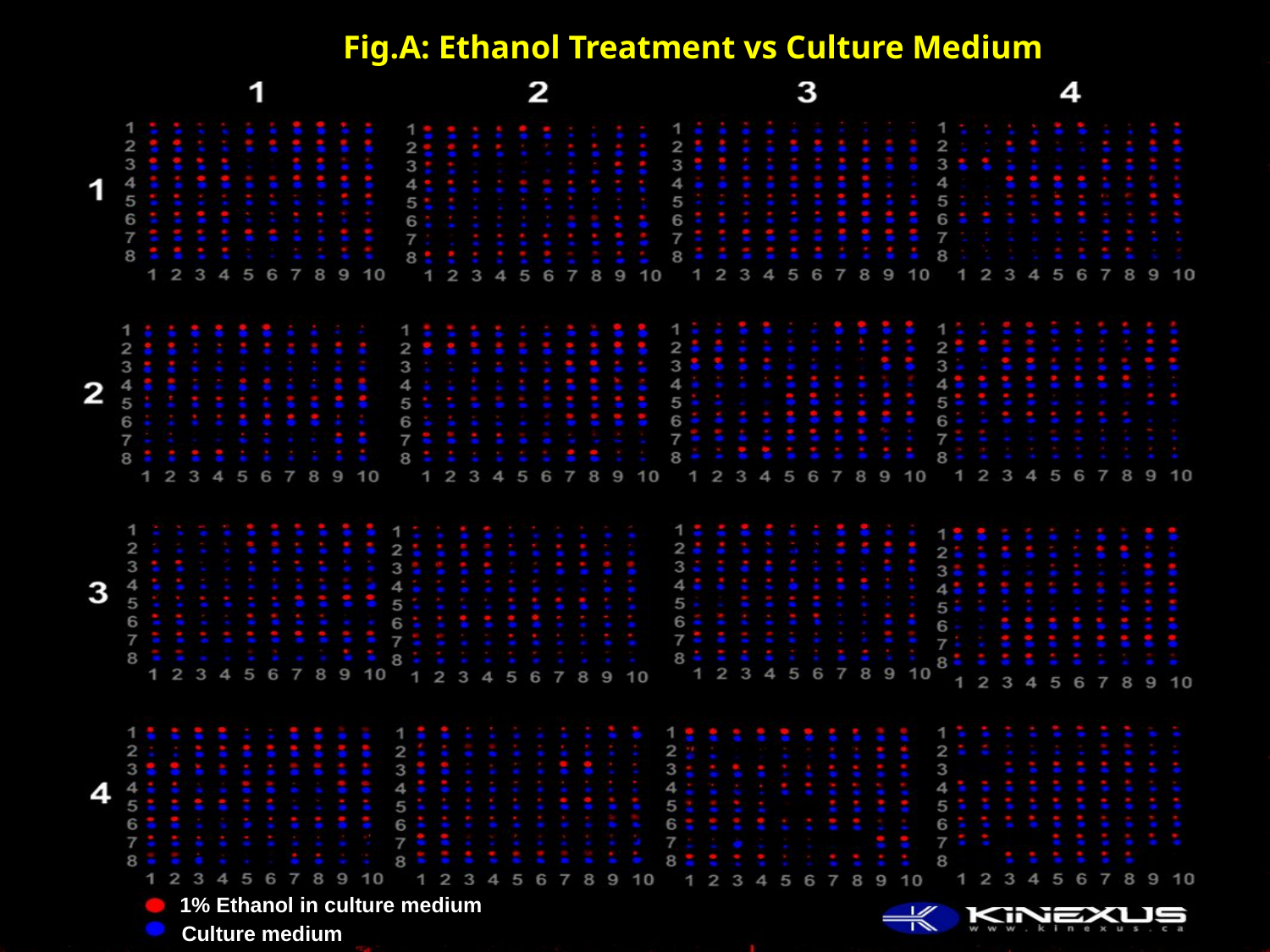

Fig.A: Ethanol Treatment vs Culture Medium
1% Ethanol in culture medium
Culture medium

## Slide 2
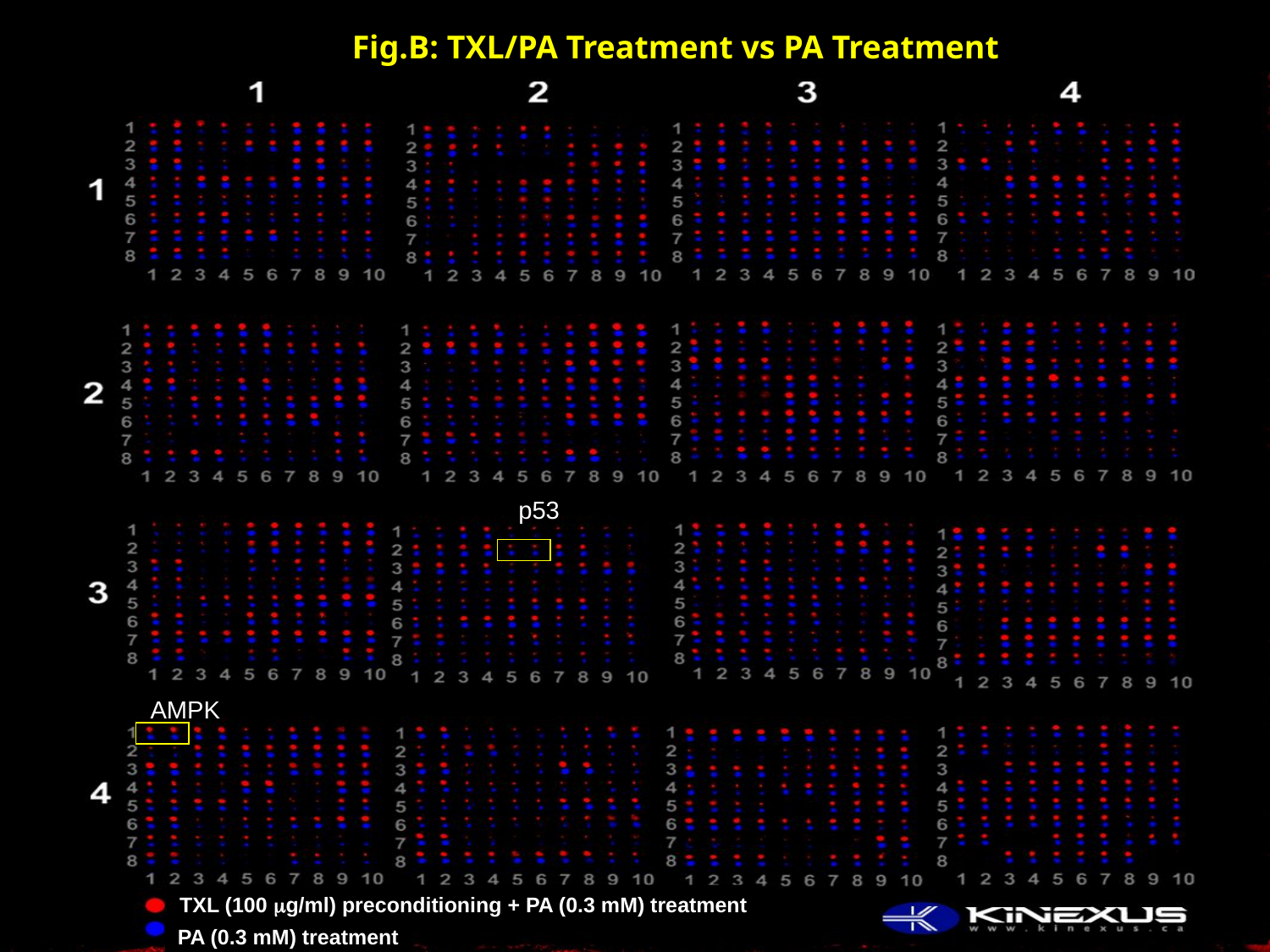

Fig.B: TXL/PA Treatment vs PA Treatment
#
p53
AMPK
TXL (100 μg/ml) preconditioning + PA (0.3 mM) treatment
PA (0.3 mM) treatment
